# Editorial: Developing medicinal plant extracts commercially: The importance of extraction and fractionation

**DOI:** 10.3389/fphar.2023.1140783

**Published:** 2023-01-23

**Authors:** Thomas Brendler, Lee Suan Chua, Luqman Chuah Abdullah, Mun Fei Yam

**Affiliations:** ^1^ Plantaphile, Collingswood, NJ, United States; ^2^ Traditional Medicinals Inc, Rohnert Park, CA, United States; ^3^ Department of Botany and Plant Biotechnology, University of Johannesburg, Johannesburg, South Africa; ^4^ Department of Bioprocess and Polymer Engineering, Faculty of Chemical and Energy Engineering, Universiti Technologi Malaysia, Johor Bahru, Malaysia; ^5^ Institute of Tropical Forestry and Forest Products, Universiti Putra Malaysia, Selangor, Malaysia; ^6^ School of Pharmaceutical Sciences, Universiti Sains Malaysia, Minden, Pulau Pinang, Malaysia

**Keywords:** extracts, fractions, medicinal plant, commercialiazation, functional properties

The use of botanical drugs is integral to healthcare practices of indigenous populations. From there stems–combined with a growing interest in and popularity of using botanical medicines and supplements in everyday healthcare and wellbeing–the steady increase in demand for readily available herbal products that are efficacious, safe and of high quality (Smith et al., 2022). Botanical medicines and supplements are manufactured into a variety of dosage forms, ranging from traditional infusions and tinctures, powders, gummies, capsules and tablets all the way to sophisticated delivery systems to enhance bioavailability and/or determine time and location of release, such as inhalates or orodispersible films and melts. Additionally, botanicals are incorporated into foods, cosmetics, and personal care products.

Methods of extraction, concentration, purification, and isolation have been an integral part of botanical medicine since its onset. Along with the increase of knowledge and understanding of the phytochemical and phytopharmaceutical properties of botanical ingredients, they have developed from simple infusions, decoctions and macerations into complex processes involving multiple steps and solvents. Indeed, with a better understanding of the characteristics of single actives or matrices thereof, suitable processing methods such as extraction and fractionation utilizing appropriate solvent systems have gained upmost importance.

Herein also lies the danger for emergence of a disjunct between the interests of academia, industry, healthcare regulators and the consumer: academia must be careful not to conduct research for research’s sake and create output that–while novel and groundbreaking–is challenging to incorporate into products or communicate to the consumer. Study of effects or compounds outside the realm of traditional uses or to address severe disease states may serve as an example here, as it puts the onus of translating results of preclinical research and conducting clinical research sufficient both in quantity and quality to meet the requirements of healthcare regulations on (pharmaceutical) manufacturers, not to forget building sustainable supply chains, which can easily become economically unobtainable or simply not practical, especially if the resulting product would be of a narrow therapeutic spectrum. This statement, however, should not be misconstrued as dismissive of cutting-edge research of compounds, their effects, and their purification, isolation and even synthetization, but rather serve as a reminder, just as much to industry, that innovation needs to include collaboration from an early stage onward in order to create botanical products that address and meet market demands (David et al., 2015).

The composition of herbal extracts, fractions, or isolates, as well as their bioavailability, very much depend on the processing methods applied. Conversely, the suitability of an extract for the desired dosage form and finished product, such as hygroscopicity, heat stability, and thus, e.g., the need for excipients, must be taken into account when creating and investigating botanical ingredients for commercial applications.

Even though the editors would have welcomed submissions from the extract manufacturing and processing industry to have been more numerous, this Research Topic provides a good perspective and cross section of the entire gamut of topics, aspects, and issues pertaining to the making and use of botanical extracts and isolates. Specifically, it covers.

• Elucidation of novel activities through fractionation of traditional medicines (https://www.frontiersin.org/articles/10.3389/fphar.2022.963160/full)
• Tools to examine functional properties of compounds obtained from plant extracts (https://www.frontiersin.org/articles/10.3389/fphar.2022.980066/full)
• Processing methods impacting extract properties (https://www.frontiersin.org/articles/10.3389/fphar.2022.1007746/full, https://www.frontiersin.org/articles/10.3389/fphar.2022.1003209/full)
• Extract properties determining suitability for dosage forms (https://www.frontiersin.org/articles/10.3389/fphar.2022.1013340/full)
• Analytical methods for the determination of authenticity of botanical extracts in finished products (https://www.frontiersin.org/articles/10.3389/fphar.2022.925298/full, https://www.frontiersin.org/articles/10.3389/fphar.2022.931203/full)
• Labeling of botanical extracts in finished products from a regulatory perspective (https://www.frontiersin.org/articles/10.3389/fphar.2022.981978/full)

Specifically (https://www.frontiersin.org/articles/10.3389/fphar.2022.963160/full) speaks to the pigmentation effect of *Epimedium brevicornum* Maxim. ethanolic extract and identifies epimedin B as an effective melanogenic compound. Authors of (https://www.frontiersin.org/articles/10.3389/fphar.2022.980066/full) have explored the versatility of yeast (*Saccharomyces cerevisiae*) in assessing the antioxidant and anti-aging capacities of flavonoids extracted from plant material. Authors of (https://www.frontiersin.org/articles/10.3389/fphar.2022.1007746/full) and (https://www.frontiersin.org/articles/10.3389/fphar.2022.1003209/full), investigated how harvesting, processing, drying and other processing steps affect properties and composition of extracts made from ginkgo (*Ginkgo biloba* L.) and chamomile (*Matricaria chamomilla* L.), respectively. Authors of (https://www.frontiersin.org/articles/10.3389/fphar.2022.1013340/full) discuss the impact of botanical extracts’ compounds and physical properties on their suitability of use in herbal tea formulation. The focus of (https://www.frontiersin.org/articles/10.3389/fphar.2022.925298/full) and (https://www.frontiersin.org/articles/10.3389/fphar.2022.931203/full) lies on the application of analytical methods, such as HPTLC and FTIR spectroscopy in quality assessment and the identification of adulterants in finished herbal products. Last, but not least (https://www.frontiersin.org/articles/10.3389/fphar.2022.981978/full) discusses the challenges of labelling botanical extracts as to appropriately inform manufacturers and consumers of their quality while adhering to regulatory requirements.

It is hoped that the collection of publications presented in this Research Topic will stimulate further research and collaboration between academia, industry, and regulators with the goal of developing and incorporating botanical extracts that are suitable for use, and safe and efficacious ingredients in a wide variety of botanical products.
